# Netosis and Inflammasomes in Large Vessel Occlusion Thrombi

**DOI:** 10.3389/fphar.2020.607287

**Published:** 2021-01-22

**Authors:** Stephanie H. Chen, Xavier O. Scott, Yoandy Ferrer Marcelo, Vania W. Almeida, Patricia L. Blackwelder, Dileep R. Yavagal, Eric C. Peterson, Robert M. Starke, W. Dalton Dietrich, Robert W. Keane, Juan Pablo de Rivero Vaccari

**Affiliations:** ^1^ Department of Neurological Surgery and the Miami Project to Cure Paralysis, University of Miami Miller School of Medicine, Miami, FL, United States; ^2^ Department of Physiology and Biophysics, University of Miami Miller School of Medicine, Miami, FL, United States; ^3^ Center for Cognitive Neuroscience and Aging University of Miami Miller School of Medicine, Miami, FL, United States; ^4^ University of Miami Center for Advanced Microscopy (UMCAM) and Department of Chemistry, University of Miami, Coral Gables, FL, United States; ^5^ Department of Neurology, University of Miami Miller School of Medicine, Miami, FL, United States

**Keywords:** inflammation, stroke, inflammasome, thrombus, caspase-1, ASC, neutrophil extracellular traps, NETs

## Abstract

The inflammatory response appears to play a critical role in clotting in which neutrophil extracellular traps (NETs) are the major drivers of thrombosis in acute ischemic stroke (AIS). The inflammasome is an innate immune complex involved in the activation of interleukin (IL)-18 and IL-1β through caspase-1, but whether the inflammasome plays a role in NETosis in AIS remains poorly understood. Here we assessed the levels of inflammasome signaling proteins in NETs and their association with clinical and procedural outcomes of mechanical thrombectomy for AIS. Electron microscopy and immunofluorescence indicate the presence of NETs in thrombi of patients with AIS. Moreover, the inflammasome signaling proteins caspase-1 and apoptosis-associated speck-like protein containing a caspase recruitment domain (ASC) were also present in clots associated with the marker of NETosis citrullinated histone ^3^H (CitH3). Analysis of protein levels by a simple plex assay show that caspase-1, ASC and interleukin (IL)-1β were significantly elevated in clots when compared to plasma of AIS patients and healthy controls, while IL-18 levels were lower. Moreover, multivariate analyses show that IL-1β levels in clots contribute to the number of passes to achieve complete recanalization, and that ASC, caspase-1 and IL-18 are significant contributors to time to recanalization. Thus, inflammasome proteins are elevated in NETs present in thrombi of patients with AIS that contribute to poor outcomes following stroke.

## Introduction

Stroke is the leading cause of long-term disability and the second leading cause of death worldwide. Although large vessel occlusion acute ischemic strokes (LVOS) account for approximately 40% of ischemic strokes, they are disproportionately associated with severe disability and mortality (Rennert et al., 2019). Currently, treatment options for LVOS are limited to intravenous alteplase (tPA) within 4.5 h as well as mechanical thrombectomy within 24 h of symptom onset (Powers et al., 2019). While early reperfusion has been shown to improve functional outcomes, many patients are ineligible or lack access to treatment (Fransen et al., 2014; Goyal et al., 2016a; Goyal et al., 2016b; Jadhav et al., 2018). Moreover, over half of the patients who are treated with endovascular intervention and/or tPA remain severely disabled or deceased at 90 days (Goyal et al., 2016b; Hankey, 2017). While the thrombus is the primary target of stroke treatment, little is known about the composition and pathogenesis following stroke. Previous studies attempted to use computed tomography (CT) and magnetic resonance imaging (MRI) to predict clot density (Niesten et al., 2014; Kim et al., 2015; Jagani et al., 2017). However, a further understanding of the dynamic processes of clot pathology is necessary in order to translate these findings into improved clinical treatment methods.

Cerebral thrombus histopathology reveals common components, including presence of platelets, leukocytes, and red blood cells in diverse histological and quantitative patterns (Brinjikji et al., 2017). The heterogeneity of thrombi composition is thought to be associated with thrombus origin. However, recent studies have detected extensive neutrophil extracellular traps (NETs) throughout all LVOS thrombi (Laridan et al., 2017; Ducroux et al., 2018). NETs are large extracellular web-like structures composed of decondensed chromatin lined with granular and cytosolic proteins (Brinkmann et al., 2004) that are formed in a cell death pathway known as NETosis (Gupta et al., 2010). In addition to acting as a scaffold for platelets and red blood cells, NETs have a pro-inflammatory role that is associated with thrombogenesis in the arterial and venous vasculature (Kimball et al., 2016; Laridan et al., 2019).

The inflammasome is a multiprotein complex comprised of a sensor such as a NOD-like receptor (NLR), the adaptor protein apoptosis-associated speck-like protein containing a caspase-recruitment domain (ASC) and the inflammatory cysteine aspartase caspase-1 (Govindarajan et al., 2020). We have previously shown that the NRLP1 inflammasome is activated following cerebral ischemia in rodents (Abulafia et al., 2009). In addition, numerous studies have reported inflammasome involvement in the pathogenesis of cerebral ischemia (Kastbom et al., 2015; Tong et al., 2015; Gao et al., 2017; Ismael et al., 2018; Yang et al., 2018; Kim et al., 2020). The NLRP1 inflammasome was the first inflammasome reported to play a role in cerebral ischemia (Abulafia et al., 2009). However, inhibition of the NLRP3 inflammasome with intravenous immunoglobulin has been shown to be neuroprotective is an animal model of stroke (Fann et al., 2013), and studies in NLRP3 knockout mice indicate that NLRP3 deletion results in decreased infarct volume, decreased edema and decreased permeability of the blood brain barrier (Yang et al., 2014). Moreover, inflammasome proteins in humans have been shown to be reliable biomarkers of central nervous system (CNS) injury (Adamczak et al., 2012; Kerr et al., 2018b; Perez-Barcena et al., 2020) and disease (Keane et al., 2018; Scott et al., 2020), including stroke (Kerr et al., 2018a). Thus, the inflammasome is an important regulator of the inflammatory innate immune response following stroke.

Here we isolated thrombi and plasma from patients following AIS and performed electron microscopy and immunofluorescent staining to determine the cytoarchitecture of thrombi and the composition of NETs and the inflammasome proteins in clots in this patient population.

## Material and Methods

### Participants

Between November 2018 and November 2019, we conducted a prospective study investigating thrombi retrieved from mechanical thrombectomy procedures in AIS patients admitted to Jackson Memorial Hospital/University of Miami Hospital (Table 1). All patients with age ≥18 years old who presented with acute stroke and underwent thrombectomy with retrieval of thrombus material were eligible for the study. Ethics approval was approved by the Institutional Review Board at the University of Miami (IRB 20160699), and informed consent was obtained from all patients included in this study. Patients were excluded if adequate thrombus material could not be obtained or the patient/legal representative refused to participate in the study. Patient demographics, clinical presentation, neurological exam (National Institutes of Health Stroke Scale (NIHSS)), pre-procedural imaging results, intravenous tissue plasminogen activator (IV-tPA) administration, procedural details including number of passes, thrombectomy technique used, recanalization results (Thrombolysis in Cerebral Infarction (TICI) scale, and follow-up data were collected. A total of 30 clots were obtained from patients undergoing mechanical thrombectomy. Following mechanical thrombectomy, six clots were fixed in 4% paraformaldehyde for histology and the remaining 24 clots were processed for molecular analysis. Healthy control samples were purchased from BioIVT (Hicksville, NY), and they were obtained from donors without any diagnosed disease.

**TABLE 1:** Baseline characteristics of patients who underwent mechanical thrombectomy.

**Patient and procedural characteristics (N = 30)**	
Mean age (StdDev)	70 (15)
Male (%)	18 (60%)
Median NIHSS (SEM)	16 (1.1)
IV tPA (%)	14 (46%)
Comorbidities	
Congestive heart failure (%)	3 (10%)
Atrial fibrillation (%)	14 (47%)
Coronary artery disease (%)	10 (33%)
Diabetes mellitus (%)	9 (30%)
Hyperlipidemia (%)	13 (43%)
Hypertension (%)	26 (87%)
Cancer (%)	5 (17%)
Prior stroke (%)	9 (30%)
Smoking (%)	13 (43%)
Substance abuse (%)	4 (13%)
Antiplatelet (%)	4 (13%)
Anticoagulation (%)	6 (20%)
Median mRS pre-MT	0 (0.23)
Median time LKN to recanalization (SEM)	303 min (92)
Mean # passes	1.8 (1.1)
Stentriever (%)	22 (73%)
ADAPT (%)	8 (2.7%)
TICI score	
2B (%)	2 (6.7%)
2C (%)	7 (23%)
3 (%)	21 (70%)
Hemorrhage	
rICH (%)	9 (30%)
sICH (%)	2 (6.7%)
Median mRS @ discharge	4 (0.39)
Death (%)	7 (23%)

tPA, tissue plasminogen activator; mRS, modified ranking score; MT, mechanical thrombectomy; LKN, Last known normal; ADAPT, a direct aspiration first pass technique, TICI: thrombolysis in cerebral infarction; rICH, radiographic intracranial hemorrhage; sIH, symptomatic intracranial hemorrhage.

### Thrombectomy Procedure

The mechanical thrombectomy procedures were all performed or supervised by board-certified neurointerventional experts under biplane neuroangiography (Artis Q, Siemens Healthcare, Erlangen, Germany). All patients were treated under general anesthesia, per institutional protocol. Site of access and thrombectomy technique were at the discretion of the treating physician. If an aspiration-alone technique was used, a large guide catheter was navigated into the cervical segment of the target vessel, then a microcatheter (0.027″) telescoped through the aspiration catheter (0.068″ or 0.071″) was introduced and navigated just proximal to the clot. With the aspiration pump initiated, the aspiration catheter was brought over the microcatheter to the face of the clot. The microcatheter was then removed for improved aspiration and the aspiration catheter was retracted. Moreover, if a Solumbra technique was used, a guide catheter (balloon guide or 0.088”) was brought into the cervical segment of the target vessel, a microcatheter telescoped through an aspiration catheter was introduced and navigated over a microwire past the site of occlusion. The microwire was then removed and the stent retriever was deployed through the microcatheter across the occluded segment. The microcatheter was then removed and aspiration from the aspiration catheter was initiated. The stent retriever was left in place for 5 min to encourage integration of the clot and then slowly retracted under constant aspiration into the guide catheter. Heparin was not administered during mechanical thrombectomy, although non-therapeutic doses of 1,000 IU unfractionated heparin were always added to the 1-L bags of standard 0.9% saline flushes in order to avoid catheter-associated thrombus formation.

### Immunofluorescence

For immunohistochemical procedures, six clots were fixed in 4% paraformaldehyde overnight, and then processed for paraffin embedment as described in (de Rivero Vaccari et al., 2009). Sections were then double-stained with primary antibodies rabbit anti-caspase-1 (Cat #06-503-I, EMD Millipore) or rabbit anti-ASC (amino acids 182-195, EMD Millipore) and mouse anti-Citrullinated-Histone H3 (amino acids 1-100, Abcam) followed by fluorescently labeled secondary Alexa Fluor antibodies (488 and 594) raised in goat (Invitrogen). Autofluorescence in sections was quenched using the Vector TrueVIEW Autofluorescence Quenching Kit (Vector Laboratories) according to manufacturer instructions. Sections were imaged using an EVOS FL Auto two Imaging System (ThermoFisher Scientific). Secondary antibodies alone were used as negative controls (Supplementary Figure 1).

### Immunoblotting

For immunoblot analysis of NLRP1 and ASC proteins from clots of nine different patients, protein was extracted and resolved as described in (Brand et al., 2015). Briefly, equal amounts of protein lysates (20 μg of total protein) were resolved in 4–20% Criterion TGX Stain-Free precasted gels (Bio-Rad). Protein was then transferred to polyvinylidene difluoride (PVDF) membranes (BioRad) using the Trans Blot Turbo System (BioRad). Membranes were then blocked in blocking buffer with I-Block (Applied Biosystems) diluted in phosphate buffered saline (PBS) and incubated with primary antibodies (1:1000 dilution) rabbit anti-NLRP1 (#NBP1-54899, Novus Biologicals) and rabbit anti-AIM2 (D-14, Santa Cruz) followed by incubation with anti-mouse IgG HRP-linked secondary antibodies (1:1000 dilution, Cell Signaling) and enhanced chemilluminescence using LumiGLO reagent (Cell Signaling). PVDF membranes were imaged using the ChemiDoc Touch Imaging System (BioRad).

### Transmission Electron Microscopy (TEM)

Blood clot samples were fixed in 2% glutaraldehyde in 0.05 M phosphate buffer and 100 mM sucrose. Then they were post-fixed overnight in 1% osmium tetroxide in 0.1 M phosphate buffer, followed by dehydration and embedment in a mixture of EM-bed/Araldite (Electron Microscopy Sciences). One μm-thick sections were then stained with Richardson’s stain for observation by light microscopy. One hundred ηM sections were then cut on a Leica Ultracut-R ultramicrotome and stained with uranyl acetate and lead citrate. Grids were viewed at 80 kV in a JEOL JEM-1400 transmission electron microscope. Images were captured by an AMT BioSprint digital camera.

### Scanning Electron Microscopy (SEM)

For SEM imaging, blood clot samples were fixed in 2% glutaraldehyde in 1X phosphate buffer saline (PBS) (E.M. Sciences,Inc.), post-fixed for 1 h in 1% osmium tetroxide in PBS buffer, rinsed in buffer, dehydrated through a graded series of ethanols, and dried after three changes of Hexamethyldisilazane (HMDS) (E.M.Sciences,Inc.). Samples were then coated with a 20 nm layer of palladium (Pd) in a plasma sputter coater, and imaged in a Philips XL-30 Field Emission SEM.

### Simple Plex Assays

Clots were analyzed using a four-plex assay for the protein expression of caspase-1, apoptosis-associated speck-like protein containing a caspase-recruitment domain (ASC), IL-18 and IL-1β (Protein Simple) as described in Brand *et al.* (Brand et al., 2016). Briefly, samples were diluted 50:50 in dilution buffer, and 50 μL were loaded in the respective wells of the cartridge. One ml of washing buffer was loaded in the assigned wells, and the assay was run in the Ella instrument (Protein Simple) using the Simple Plex Runner 3.5.2.20 software. Data were then processed using the Simple Plex Explorer 3.5.2.20, and further analyzed by Prism 8.0 statistical software (GraphPad Prism). Results presented correspond to the mean of samples run in triplicates.

### Statistical Analyses

Statistical analyses were carried using Prism 8 (GraphPad Prism) software. Data were tested for normality using the D’Agostino and Pearson omnibus normality test. Comparison between groups for normally distributed data were done using a Kruskal-Wallis test followed by Dunn’s multiple comparison test for data that were not normally distributed. *p*-values of significance were *p* < 0.05. Mean values of inflammatory cytokines in the clot lysate of LVOS patients were compared to plasma levels of stroke patients and healthy controls. In addition, linear and logistic regression using inflammasome protein concentration in the clot of patients and other clinical variables were done using RStudio software Version 1.2.5033 using the following packages: ggplot2, MASS, dplyr, broom, car, regclass and ROCit and with Stata 10.0 (College Station, TX). Factors predictive in univariate analysis (*p* < 0.15) were entered into a multivariate logistic regression analysis. *p*-values of ≤0.05 were considered statistically significant.

## Results

### Patients With AIS

A total of 30 clots were retrieved by mechanical thrombectomy from patients presenting with acute large vessel occlusion stroke (Table 1). Mean patient age was 70 years old and the majority of patients were male (60%). Median national institutes of health stroke scale (NIHSS) score on presentation was 16, median time from last known well to recanalization was 303 min, all patients had a thrombolysis in cerebral infarction (TICI) score of 2B or greater, 11 patients had a modified ranking scale (mRS) less than three at discharge, and 23% of patients died during the hospitalization (Zaidat et al., 2018).

### NETosis Is Present in the Clots of Patients With AIS

**FIGURE 1:**
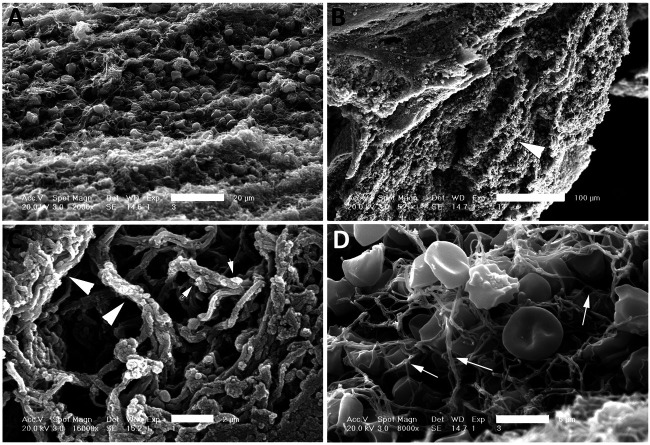
SEM of clots from AIS patients. Clots were processed for SEM indicating the presence of red blood cells **(A and D)**, fibrin (arrow heads, B and C), histones (short arrows, C) and interconnected fibers (arrows, **D)** consistent with the presence of NETs in the clots of these patients. Scale bars: **(A)** 20 μm, **(B)** 100 μm, **(C)** 2 μm, **(D)** 5 μm.

**FIGURE 2:**
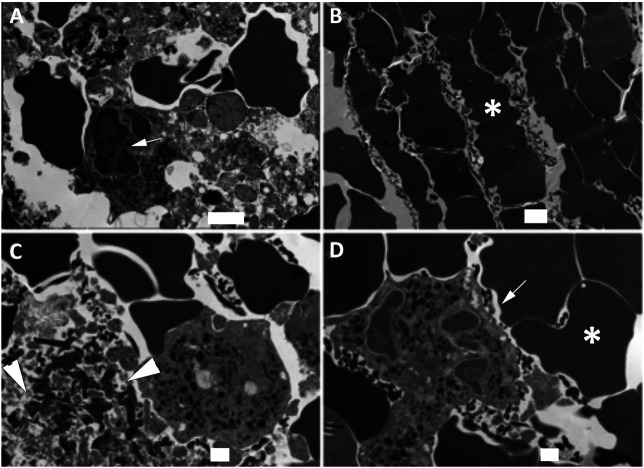
TEM of clots from AIS patients. Clots were processed for TEM indicating the presence of granulocytes (arrows, **A and D)**, red blood cells (asterisk, **B and D)**, dying neutrophils (arrow heads, C) consistent with the presence of NETs in the clots of these patients. Scale bars: **(A)** 2 μm, **(B)** 2 μm, **(C)** 1 μm, **(D)** 1 μm.

Coarse fibrin and activated platelets have been previously described in electron microscopy images of blood clots (Kawasaki et al., 2004). Isolated clots from patients that underwent thrombectomy following ischemic stroke were fixed and processed for electron microscopy procedures. Processed sections of clots from three different patients were analyzed by SEM (Figure 1) and TEM (Figure 2). A series of images were collected and the most representative images are presented in Figures 1 and 2. Accordingly, clots presented deformed red blood cells (Figures 1A and 1D), neutrophils (asterisk, 1D), interconnecting fibers (arrows, Figure 1D), fibrin (arrow heads, Figures 1B and 1C) and histones (short arrows, Figure 1C) that are consistent with the presence of neutrophil extracellular trap (NET) fibers. In addition, transmission electron microscopy analysis of the clots (Figure 2) indicate the presence of granulocytes (arrows, Figures 2A and 2D), red blood cells (asterisk, Figures 2B and 2D) and dying neutrophils (arrow heads, Figure 2C). Thus, these findings indicate that NETs are present in the clots of patients with LVOS in addition to neutrophils, deformed red blood cells, platelets, and fibrin.

### Inflammasome Proteins Are Present in NETs of Patients With AIS

**FIGURE 3:**
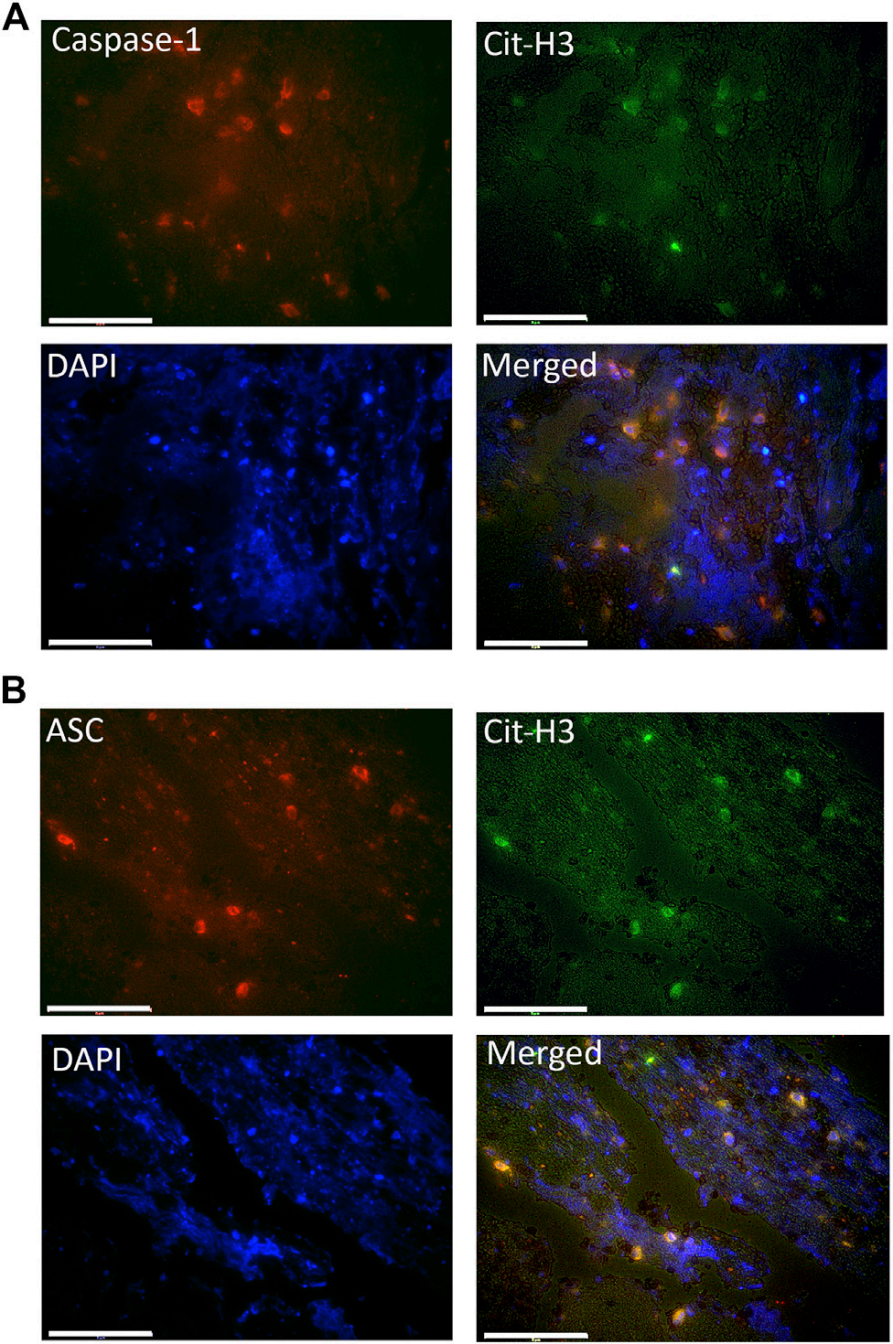
Caspase-1 and ASC are present within citrullinated-H3 structures. Fluorescent images of the clots of patients that were double-stained for Cit-H3 (green) and **(A)** caspase-1 (red) or **(B)** ASC (red). Caspase-1 and ASC positive cells were also immunorective for Cit-H3 (yellow) and nuclei were stained with DAPI (blue). Magnification: 60X. Scale bar: 75 μm.

Inflammasome signaling in neutrophils has been previously associated with the formation of NETs and NETosis activation (Chen et al., 2018). To determine if inflammasome proteins are present in NETs present in the clots of patients with AIS, we stained immunohistochemical sections with antibodies against the inflammasome signaling proteins caspase-1 and ASC, as well as citrullinated histone-3 (Cit-^3^H), a marker of NETs. (Hirose et al., 2014). Figure 3 shows that caspase-1 (green) and ASC (red) immunoreactivity were present in structures positive for CitH3, indicating that the inflammasome proteins caspase-1 and ASC are present in NETs within the clots of patients with AIS.

### Inflammasome Signaling Proteins Are Elevated in the Clots of Patients With AIS

**FIGURE 4:**
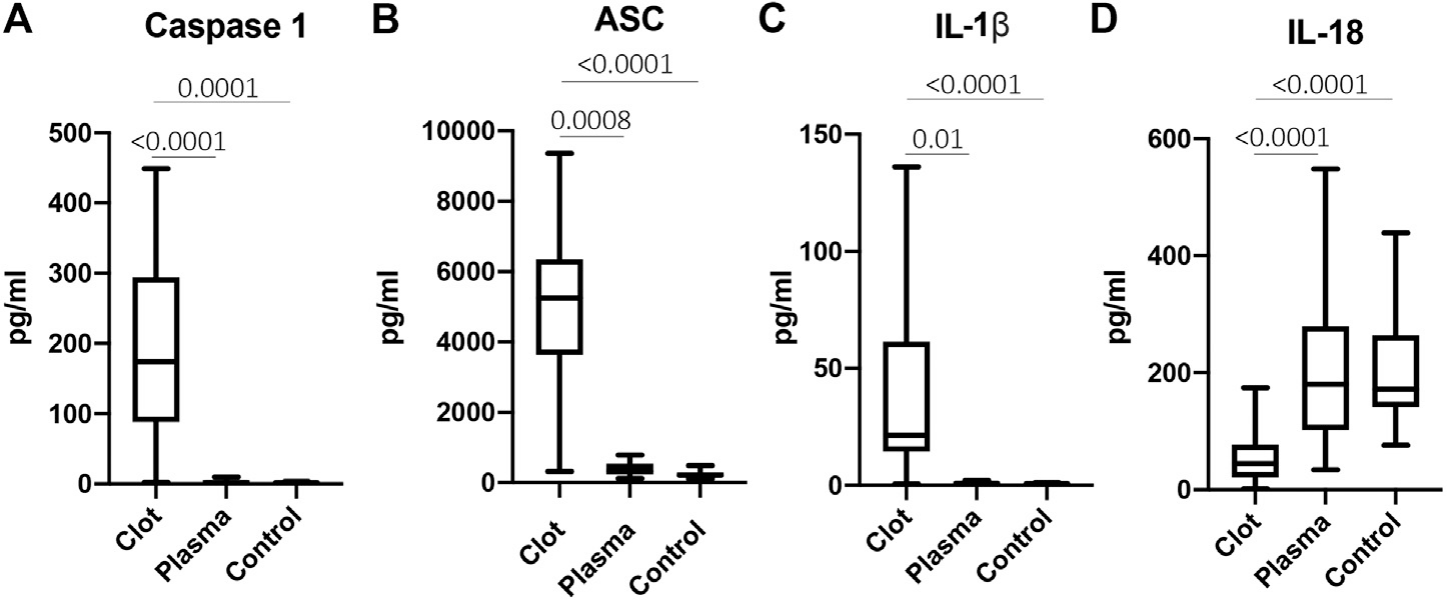
Inflammasome signaling proteins are elevated in the clots of patients with stroke. Protein levels in pg/ml of caspase-1 **(A)**, ASC **(B)**, IL-1β **(C)** and IL-18 **(D)** in the clots of patients with AIS as well as plasma of stroke patients (plasma) and from healthy controls (control). Caspase-1: N = 26 clot, N = 13 plasma stroke patients, N = 39 plasma healthy control; ASC: N = 26 clot, N = 11 plasma stroke patients, N = 8 plasma healthy control; IL-1β: N = 19 clot, N = 5 plasma stroke patients, N = 15 plasma healthy control; IL-18: N = 25 clot, N = 15 plasma stroke patients, N = 39 plasma healthy control a. Box and whiskers are shown for the 5th and 95th percentile. *p*-value of significance <0.05. All groups were compared by a Kruskall-Wallis test followed by Dunn’s multiple comparison test.

Inflammasome proteins have been previously shown to be elevated in the serum and extracellular vesicles of patients with stroke (Kerr et al., 2018a). To determine if inflammasome signaling proteins were elevated in the clots of patients with stroke, we obtained protein lysates from the clots of patients with AIS and analyzed the protein levels of caspase-1, ASC, IL-1β and IL-18 compared them to the plasma of stroke patients (plasma) and healthy controls (control). Caspase-1 (Mean values = thrombi: 191 pg/ml, plasma: 3.26 pg/ml, healthy control: 2.09 pg/ml) (Figure 4A), ASC (Mean values = thrombi: 5,039 pg/ml, plasma: 386.9 pg/ml, healthy control: 243.5 pg/ml) (Figure 4B) and IL-1β (Mean values = thrombi: 39.82 pg/ml, plasma: 0.92 pg/ml, healthy control: 0.68 pg/ml) (Figure 4C) were elevated in the clot when compared to plasma in AIS and healthy controls; whereas IL-18 protein levels were lower in the clot than in the plasma of healthy controls and AIS patients (Mean values = thrombi: 53.31 pg/ml, plasma: 201 pg/ml, healthy control: 200.2 pg/ml) (Figure 4D). Importantly, the levels of caspase-1, IL-1β and IL-18 measured were total protein values and do not differentiate between the pro-forms and the cleaved forms of these proteins. Taken together, these findings indicate that acute inflammasome signaling protein expression is higher in the clots of AIS patients consistent with higher levels of IL-1β.

### NLRP1 and AIM2 Are Present in the Clots of Patients With AIS

**FIGURE 5:**
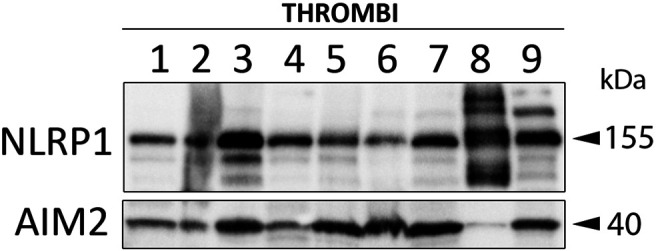
NLRP1 and AIM2 are expressed in the clots of patients with AIS. Immunoblot analysis of clots from nine AIS patients that were blotted for NLRP1 and AIM2.

In rodents, the NLRP1 inflammasome has been previously shown to contribute to the innate immune inflammatory following thromboembolic stroke (Abulafia et al., 2009). To determine which NLR sensor molecules were present in the clot of patients with stroke, we immunoblotted samples for NOD-like receptor protein-1 (NLRP1) and Absent in Melanoma-2 (AIM2); two receptors that form protein-protein interactions with caspase-1 and ASC to form an inflammasome complex. Accordingly, NLRP1 and AIM2 were present in the clots of nine patients with stroke (Figure 5). Interestingly, patient eight differed in its expression of NLRP1 *vs.* AIM2, in which NLRP1 showed laddering of the protein (Levinsohn et al., 2012), that may indicate cleavage of NLRP1 or post-translational modifications during inflammasome activation of this sensor molecule that do not occur in activation of AIM2 in the clot of the same patient.

### IL-1β and TICI Score Contribute to the Number of Passes to Achieve Recanalization

The number of passes needed to achieve complete recanalization is known to correlate with better outcomes after stroke, so that the less passes needed, the better the outcomes (Zaidat et al., 2018). Here we developed a logistic regression model using inflammasome protein levels to explain the influence of inflammasome proteins in clots to the number of passes. Our data indicate that IL-1β positively contributes (*p* = 0.049) to the number of passes whereas the TICI score, as expected, negatively contributes (*p* = 0.016) to the number of passes (Table 2). Thus, in regards to the number of passes and inflammasome signaling proteins, as IL-1β protein levels in the clot increase, so do the odds of increasing the number of passes to achieve complete recanalization as well.

**TABLE 2:** Logistic regression output of factors influencing number of passes.

Factors influencing Number of Passes			
	Estimate	Std. Error	*p*-Value
IL-18	0.045	0.030	0.131
IL-1β	0.019	0.010	0.049*
TICI	−5.353	2.221	0.016*
Smoking	−0.951	0.835	0.255

TICI, Thrombolysis in Cerebral Infarction. ^∗^p < 0.05.

### Inflammasome Proteins Affect the Last Known Normal (LKN) Time to Recanalization

Recanalization is a main determinant of patient outcomes (Yeo et al., 2013). Here we fit a linear regression model to explain what factors contribute to the LKN time to recanalization using the protein levels of ASC, caspase-1, IL-18 and IL-1β as well as tissue plasminogen activator (TPA), Body Mass Index (BMI), Coronary artery disease (CAD) and whether patients had diabetes or not (Table 3). The model indicates that in the clots, caspase-1 (*p* = 0.016) and IL-18 (*p* = 0.043), CAD (*p* = 0.004) and DM (*p* = 0.037) positively contributed to the LKN time to recanalization, whereas ASC (*p* = 0.041) presented a negative correlation to the LKN time to recanalization outcome. Together, based on the adjusted R-squared, this model explained 41% of the LKN time to recanalization.

**TABLE 3:** Linear regression output for factors affecting LKN time to recanalization.

Factors Influencing LKN Time to Recanalization (min)			
	Estimate	Std. Error	*p*-Value
ASC	−0.195	0.087	0.041*
Caspase-1	3.872	1.429	0.016*
IL-18	1.777	0.805	0.043*
IL-1β	1.904	1.094	0.102
TPA	−145.342	208.956	0.497
BMI	−31.165	15.832	0.068
CAD	888.713	264.213	0.004*
DM	601.35	263.286	0.037*

tPA, tissue plasminogen activator; BMI, body mass index; CAD, Coronary artery disease; DM, Diabetes Mellitus. ^∗^p < 0.05.

## Discussion

**FIGURE 6:**
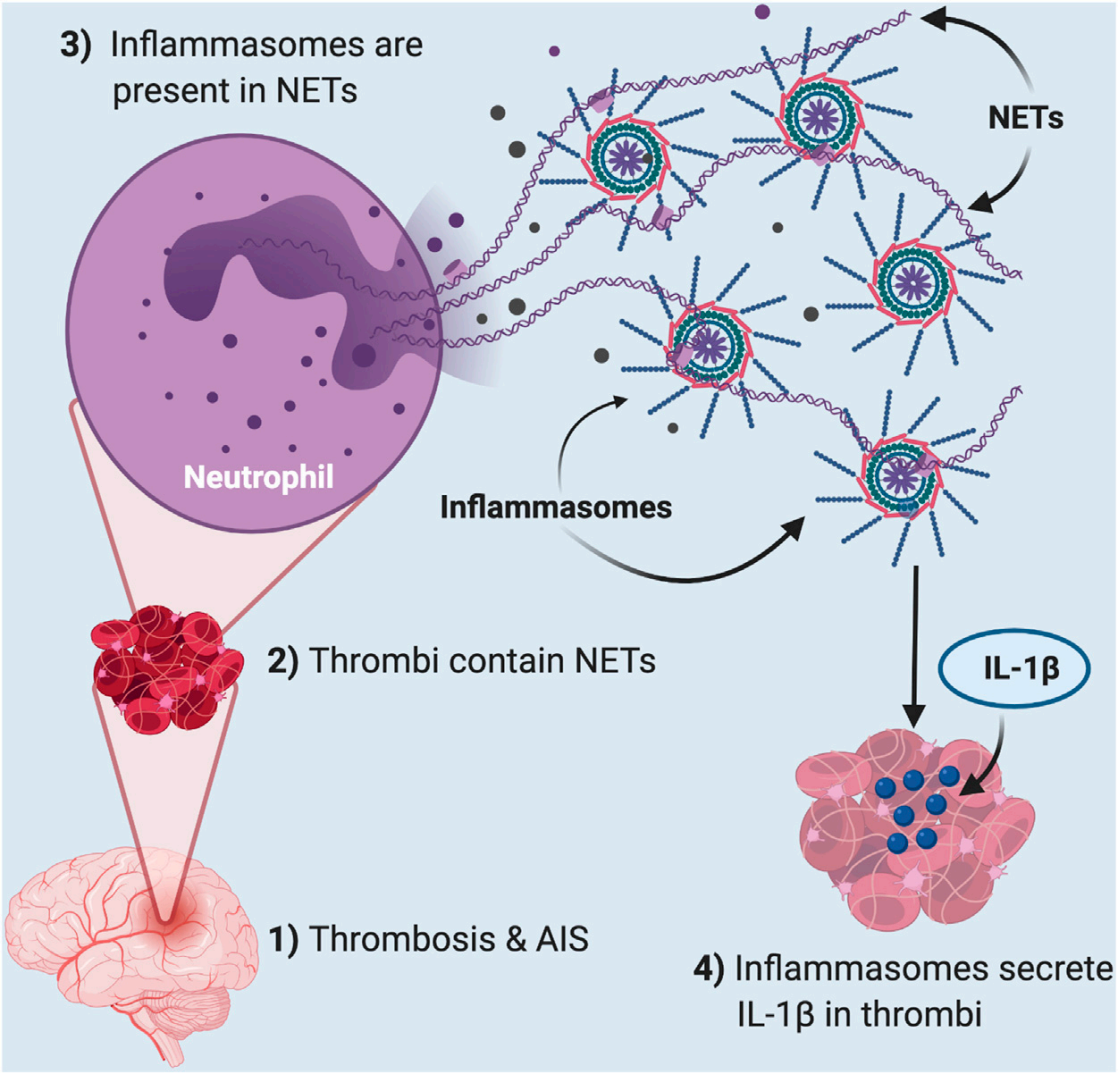
Thrombosis in acute ischemic stroke induces inflammasome activation in NETs present in clots. In AIS (1), thrombi (2) contain neutrophil extracellular traps (NETs) that contain inflammasomes (3). These inflammasomes are responsible for the released IL-1β in the thrombi of these patients (4).

Inflammatory mechanisms initiate clotting, decrease natural anticoagulant activity, and impair the fibrinolytic system (Levi et al., 2004). Recent studies have shown that NETosis plays a role in thrombosis in stroke, suggesting that NETs play a critical role in inflammatory and thrombotic disorders (Ducroux et al., 2018). Our earlier study found elevated levels of inflammasome proteins in serum of stroke patients (Kerr et al., 2018a). Here we extend these observations and show that inflammasome proteins are present in cerebral stroke thrombi that localize with NETs (Figure 6). These findings are consistent with previous studies that show that inflammasome activation is critical for NETosis (Westerterp et al., 2018). Thus, it appears that inflammasome activation contributes to the pathophysiology of cerebral stroke thrombi that associate with NETs and that the level of inflammasome proteins is predictive of outcome.

Neutrophils are key cells of the immune system capable of phagocytosis, degranulation and release of NETs (Papayannopoulos, 2018). NETs are extracellular structures comprised of cytosolic and granule proteins intertwined with scaffolds of chromatin that has been decondensed (Brinkmann et al., 2004). NETs become extracellular by the cell death process of NETosis (Fuchs et al., 2007). NETs have been shown to form in vein occlusive events such as deep vein thrombosis and maybe associated with the hypoxia that induces NETosis (Brill et al., 2011; Etulain et al., 2015).

NETs quantity and content is correlated with endovascular thrombectomy procedure length as well as number of passes required to remove the clot (Ducroux et al., 2018). However, the pathogenesis of activation of NET formation in cerebral thrombi remains unknown. In mouse models of atherosclerosis, cholesterol crystals induce inflammation by activating macrophage and neutrophil inflammasomes (Warnatsch et al., 2015). Inflammasomes are cytoplasmic multiprotein complexes containing caspase-1, the adaptor protein ASC and an NLR or ALR sensor molecule (e.g., NLRP1, AIM2). Inflammasomes process the pro-inflammatory cytokines IL-1β and IL-18 into their active forms (de Rivero Vaccari et al., 2014; de Rivero Vaccari et al., 2016). In neutrophils, activated caspase-1 or caspase-11 cleave gasdermin-D (GSDM-D), which leads to pyroptosis and NETosis (Chen et al., 2018; Chen et al., 2020). Moreover, NETs are downstream of neutrophil inflammasome activation (Chen et al., 2018; Sollberger et al., 2018; Westerterp et al., 2018).

The AIM2 inflammasome in the CNS is activated by double stranded DNA (dsDNA) (Adamczak et al., 2014), and dsDNA is present in NETs associated with atherosclerotic lesions. These findings suggest that NETs are capable of activating inflammasomes. In addition, our findings show that AIM2 is present in thrombi of patients with AIS. Moreover, previous findings also indicate that inflammasomes can also promote NET formation in a process that relies of caspase-11 and gasdermin-D cleavage (Chen et al., 2018; Sollberger et al., 2018). Furthermore, NETs have been shown to activate NLRP3 inflammasome (Kahlenberg et al., 2013). However, in this study we were unable to detect by immunoblotting procedures NLRP3 in thrombi (data not shown). However, NLRP1 and AIM2 were readily identified in clots using the same methodologies (Abulafia et al., 2009). Unlike other NLRs, such as NLRP3, NLRP1 is cleaved as part of its activation process (Levinsohn et al., 2012). Interestingly, immunoblots of a thrombus from one patient (patient 8) showed NLRP1 laddering, indicating NLRP1 cleavage or post-translational modifications. However, that same patient showed very low levels of AIM2, another inflammasome complex involved in inflammation and pyroptosis (Adamczak et al., 2014). Although beyond the scope of this project, it is possible that AIM2 is the active inflammasome in the clots of the other patients, and in patient 8, the inflammasome that was activated was the NLPR1 inflammasome instead, which would explain the lack of cleaved NLRP1 products in the clots of the other patients. Moreover, another possibility is that multiple inflammasomes may be activated in the same clots, thus producing an even more heightened innate immune response since clots from other patients such as patient three had more cleavage fragments of NLRP1 as well as higher expression of AIM2 than in other patients e.g., patient 6 (for NLRP1) or patient 4 (for AIM2). Future studies are needed to determine the role of different NLRs and AIM-2 like receptors (ALRs) in the clots of stroke patients.

Our findings show that IL-1β is significantly elevated in cerebral thrombi in contrast to the plasma of stroke patients and healthy controls. IL-1β has a fundamental role in inflammation and coagulation (Wada et al., 1991; Danton and Dietrich, 2003). Previous studies have found that IL-1β contributes to slow progressive chronic conditions such as atherosclerosis, diabetes, osteoarthritis and acute ischemic processes, including myocardial infarction and stroke (Gabay et al., 2010; Kerr et al., 2018a). In particular, IL-1β down-regulates thrombomodulin and impairs protein C activity, thus acting as a procoagulant. Furthermore, platelets express IL-1-R1 receptor and the presence of its ligand, IL1β, results in platelet hyperactivation and clumping (Bester and Pretorius, 2016). Consistent with the hyperinflammatory response in the clot are our findings of neutrophil composition and deformed red blood cells within the clot as shown by electron microscopy. Thus, inflammasome activation in cerebral thrombi may lead to further clot propagation and stabilization, and hinder breakdown by the body’s natural anticoagulant processes.

In contrast, IL-18 was significantly decreased in cerebral thrombi as compared to plasma of healthy controls. IL-18 is an important immunoregulatory cytokine that is involved in the production of IFN-γ and T cell polarization as well as increasing cell adhesion molecules, nitric oxide synthesis, and chemokine induction (Dinarello et al., 2013). However, unlike IL-1β, precursor IL-18 is constitutively expressed by whole blood cells and epithelial cells (Dinarello et al., 2013), and may explain our finding that patients had lower levels of IL-18 in cerebral thrombi as compared to plasma. It is also possible that in the clot there is compensatory mechanism in which as IL-1β levels increase, the levels of IL-18 decrease in order to modulate the exacerbated inflammatory response present at the clot site.

High resolution SEM and TEM showed that NETs are structures comprised of stretches of globular proteins. These proteins are released into the extracellular matrix by activated or dying neutrophils as a result of damage or infection (Kessenbrock et al., 2009). A key protein involved in NETosis is CitH3, characteristic of decondensed chromatin structures and hypercitrullination of histone H3 by peptidylarginine deiminase 4 (PAD4) (Fuchs et al., 2007). In support of this observation, we found CitH3 within clot structures, which contained the inflammasome proteins caspase-1 and ASC, indicating heightened inflammasome activation in NETosis in thrombi following AIS.

The activation of IL-1β and other proinflammatory cytokines recruit myeloid cells to the vascular endothelium to initiate remodeling and perpetuate inflammation (Duewell et al., 2010). In more advanced stages of the disease, cytokines destabilize atherosclerotic plaques by promoting apoptosis and matrix degradation. In patients with carotid plaques, elevated levels of IL-1β, IL-6, IL-8, IL-12 p70, IFN-γ, TNF and caspase-3 are significantly higher in rupture-prone post bifurcation segments of the plaque, suggesting a prominent inflammatory role in creating cerebral emboli (Caparosa et al., 2019). Thus, inflammasome activation may influence NETs and coagulation at the site of cerebrovascular occlusion, thus affecting thrombectomy outcomes, inflammasome activation may also be a product of emboli formation.

Importantly, NETs in thrombi may act as molecular filters for a variety of proteins, including inflammasome proteins. However, whether those proteins are catalytically active or capable of carrying out other roles in the inflammasome pathway is presently under investigation in our laboratory. Future studies are needed to analyze the role of inflammasome activation in neutrophils isolated from thrombi of AIS on the cell mediated processes of NETosis and pyroptosis. It is also critical to establish how these processes affect the inflammatory milieu in the thrombus microenvironment and in the systemic inflammatory response after cerebral ischemia. However, we have previously shown secreted inflammasome proteins caspase-1, ASC, IL-1β and IL-18 correlate with poorer outcomes in a variety of diseases or conditions of the nervous system and periphery, suggesting that these secreted inflammasome proteins are functional in inflammasome signaling (Kerr et al., 2018a; Kerr et al., 2018b; Keane et al., 2018; Forouzandeh et al., 2020; Perez-Barcena et al., 2020; Scott et al., 2020), suggesting an important role in disease- or trauma-related inflammatory pathological processes.

Our study is limited by the small number of samples as well as the significant heterogeneity that exists between patients and providers. There is not a standardized mechanical thrombectomy technique and both tools and technique remain at the discretion of the provider. Furthermore, there is a selection bias wherein clot samples are only available in patients who had at least partial success in clot removal. Thus, there are very few patients in the cohort where at least partial successful recanalization was not achieved. Additionally, there is selection bias for techniques wherein the clot could be preserved such as stentriever as opposed to aspiration alone. Nonetheless, our regression analyses indicate that inflammasome proteins in thrombi from AIS patients were associated with a greater number of thrombectomy passes in order to achieve complete recanalization, which is consistent with a longer time to achieve reperfusion and poorer outcomes in AIS patients. While, clot properties such as size, density, and strength were not obtained for this study, future studies are necessary to assess the association of inflammasome concentration with physical clot properties.

Taken together, our results provide evidence for inflammasome activation and NETosis in cerebral thrombi as well as a significant role of inflammasome proteins in contributing to poorer outcomes in patients after stroke. Therefore, an improved mechanistic understanding of the role of inflammasomes in NETosis will help in the development of therapies to treat thrombotic and inflammatory disorders, including stroke.

## Data Availability Statement

The raw data supporting the conclusions of this article will be made available by the authors, without undue reservation.

## Authors Contributions

SC, XS, YM, VA, PB, DY, EP, RS, and JdR performed the experiments. SC, DY, EP, RS, WD, RK, and JdR contributed to the study design. SC, XS, YM, VA, and PB contributed to data acquisition and analysis. JdR also contributed to data acquisition and analysis. All authors contributed to preparation of the manuscript.

## Funding

This research received grant funding from the Robert J. Dempsey Cerebrovascular Research Award to SC and JdR, an R01 grant from the NIH/NINDS to RK and JdR (R01NS113969-01) and a James and Ester King Biomedical Research Program grant from the State of Florida (7JK03) to WD. RS research is supported by the NREF, Joe Niekro Foundation, Brain Aneurysm Foundation, Bee Foundation, and by the NIH (R01NS111119-01A1) and (UL1TR002736, KL2TR002737) through the Miami Clinical and Translational Science Institute, from the National Center for Advancing Translational Sciences and the National Institute on Minority Health and Health Disparities. Its contents are solely the responsibility of the authors and do not necessarily represent the official views of the NIH.

## Conflict of Interest

JdR, RK and WD are co-founders and managing members of InflamaCORE, LLC and have patents on inflammasome proteins as biomarkers of injury and disease as well as on targeting inflammasome proteins for therapeutic purposes. JdR, RK and WD are scientific advisory board members of ZyVersa Therapeutics. EP is a consultant for Stryker Neurovascular, Penumbra, Medtronic Neurovascular and Cerenovus, as well as a stockholder for RIST Neurovascular. DY is a consultant for Medtronic Neurovascular, Cerenovus, Rapid Medical and Neuralanalytics. RS has consulting and teaching agreements with Penumbra, Abbott, Medtronic, InNeuroCo and Cerenovus.

The remaining authors declare that the research was conducted in the absence of any commercial or financial relationships that could be construed as a potential conflict of interest.
