# Supplementary figures and images for: Netosis and Inflammasomes in Large Vessel Occlusion Thrombi
Source: Front Pharmacol. 2021 Jan 22;11:607287. doi: 10.3389/fphar.2020.607287 (PMC7868597; doi:10.3389/fphar.2020.607287)

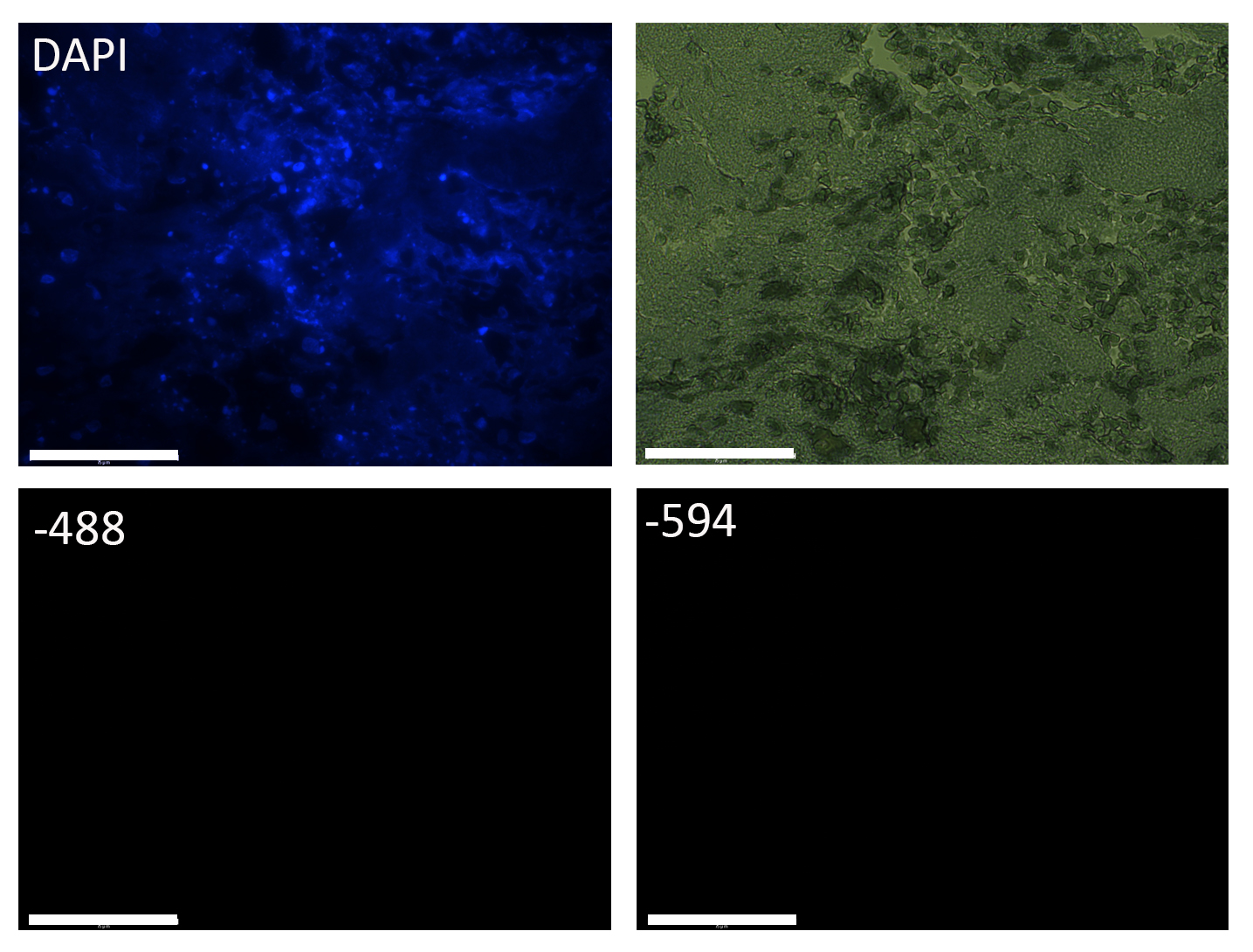

Supplement: Supplementary file 1 [file image1.jpeg]
